# Prevalence, pattern and predictors of elder abuse in rural communities of Edo State, Nigeria

**DOI:** 10.4314/gmj.v59i2.4

**Published:** 2025-06

**Authors:** Fatelyn I Okakah, Simeon N Awunor, Oluwaseun E Daramola, Danny A Asogun, Charles O Aluede, Ejiroghene C Ucho

**Affiliations:** 1 Department of Community Medicine, Irrua Specialist Teaching Hospital, Irrua, Edo State, Nigeria; 2 Department of Community Medicine, Delta State University, Abraka, Nigeria; 3 AiicoMultishield Healthcare Limited (HMO), Abuja, Nigeria; 4 Ambrose Alli University, Ekpoma, Nigeria

**Keywords:** Elder abuse, Prevalence, Pattern, help-seeking behaviour, Rural

## Abstract

**Objective:**

This study aimed to investigate the prevalence, pattern, and predictors of elder abuse in two rural communities in Edo State, Nigeria.

**Design:**

A cross-sectional study.

**Setting:**

Individuals residing in rural communities in Edo state were studied.

**Participants:**

Two hundred and thirty-two participants aged ≥ 60 years.

**Main outcome measures:**

The prevalence and pattern of elder abuse, and predictors of abuse.

**Results:**

The mean age of participants was 73.2±9.1 years, and the prevalence of elder abuse was 79.3%. The pattern of abuse observed was neglect (73.4%), financial abuse (64.1%) and emotional abuse (53.8%), with many of the perpetrators being children of the victims. Risk factors associated with abuse in this study included sex (p = 0.009), marital status (p = 0.028), employment status (p = 0.002), educational qualification (p = 0.001), living arrangement (p = 0.001), financial status (p = 0.017), and dependency level (p = 0.015). The top barriers to help-seeking by the elderly were stigmatisation (24.4%), fear of retaliatory assault (19.3%) and abandonment (19.4%) respectively.

**Conclusion:**

Elder abuse is prevalent in the study area, with neglect being the most typical form of abuse and children of the victims are the major perpetrators. Consequently, concerted efforts to respect and care for the elderly should be directed by all stakeholders at the International, Federal, State, and Local Levels (including the community and family).

**Funding:**

None declared

## Introduction

Elder abuse is rapidly becoming a social and public health problem globally.^1^,^2^ Elder abuse is a single or repeated act or lack of appropriate action, occurring within any relationship where there is an expectation of trust which causes harm or distress to an older person.^3^

There is a rise in the ageing population, and the number of older persons is increasing due to global improvements in healthcare and increasing life expectancy. With this, there is also a rise in elder abuse. In the coming decades, there will be a dramatic increase in the population of the elderly and, invariably, the number of those at risk of abuse. It is estimated that by 2025, one million people will reach the age of 60 every month.

By 2050, the global population of those aged 60 years and above is expected to more than double, from 900 million in 2015 to approximately 2 billion, with 80% of these individuals projected to be from the developing world.^4^ However, the current situation and future outlook suggest that the quest for better life and greener pastures will continue to result in mass rural-urban migration of many young adults, leaving older parents in the rural areas.^5^

Elder abuse is a global health problem with varying prevalence. However, in many countries, awareness about its existence is just developing as it is a concealed problem, hidden from public view and cloaked under the shroud of family secrecy.^6^

Generally, five types of elder abuse have been identified, and these include 1) physical abuse i.e. such physical assaults, physical restraints and force feeding, 2) emotional/psychological abuse i.e. verbal harassment or humiliation and other behaviours that cause mental anguish, pain or distress on an elderly person, 3) Financial/material abuse or exploitation, 4) sexual abuse i.e. rape and coerced nudity, and any sexual assault, 5) abandonment, neglect or failure to provide adequate care.^7^-^10^

This study assessed the prevalence, pattern and predictors of elder abuse in rural communities of Edo State, Nigeria. Findings from this study will fill the existing gap in the literature on elder abuse in Nigeria, as well as provide information to all relevant stakeholders on the burden of the problem and the need for improved social security for the elderly.

## Methods

This was a community-based cross-sectional study carried out among the elderly (male and female of 60 years and above) between May 2019 and May 2020 at two rural towns: Eror, in Esan Northeast LGA, with an estimated population of 8,837,^11^ and Ogba, on the outskirts of Oredo LGA, with an estimated population of 12,506.^12^. These towns were selected using a multistage sampling technique. Firstly, two of the three senatorial districts that make up Edo state were selected using simple random sampling by ballot. Edo South and Edo Central were selected, which comprised seven (7) and five (5) Local Government Areas (LGAs) respectively. One LGA was further selected from each senatorial district. Thus, Oredo LGA was selected from Edo South and Esan Northeast LGA from Edo Central. A rural community was therefore chosen randomly from each Local Government Area (LGA) by ballot.

The sample size consisted of 232 respondents, calculated using the Cochran formula.^13^ The inclusion criteria were any consenting elderly person (male and female) in the selected study areas. Elderly persons with cognitive impairment, those with communication difficulties (e.g., those with speech defects, deafness, and blindness), and those who were physically too ill to participate were excluded from the study.

A multistage sampling technique was used to select respondents for the study. Data was collected using pretested, interviewer - administered semi-structured questionnaire adapted from a standardised questionnaire on elder abuse and neglect developed by the World Health Organization (WHO, 2008); and a mini mental state examination (MMSE) guide adapted from the mini mental state examination by the London Alzheimer society to assess the cognitive ability of the respondents by testing their orientation to time, place, immediate recall, object identification and simple calculation ability.

The questionnaire obtained information on basic Sociodemographic characteristics of respondents, Awareness, knowledge and perception of elder abuse, Prevalence and pattern of elder abuse and Factors associated with abuse. Nigerian Pidgin, a common means of communication, was used to interpret the questionnaire for most respondents; however, in some cases, the local language was used when necessary. Respondents' ages were determined by asking them about their ages or by estimation (if unknown to them) from historical events like the 1947 eclipse of the sun, Nigeria's independence (1960) or the civil war (1967-1970). Due to the sensitivity of the topic and the confidentiality required, help for interpretation by community members was not sought. Thus, any person with communication difficulties was excluded from the survey.

Positive answers to assess the various types of abuse attracted a mark each, while a negative answer scored zero. A score of one or more in any category of the pattern of abuse was taken as a positive experience for that type of abuse. Thereafter, frequencies and percentages were derived for the various patterns of abuse. Prevalence was calculated as the number of individuals who had experienced any abuse in the past year before the study, divided by the total number of respondents interviewed, and expressed as a percentage (x100). Awareness of elder abuse was assessed with a “Yes” and “No” response to a question asked (Have you ever heard the term elder abuse?), and the proportion of respondents with a positive answer was estimated. A level of awareness greater than 50% was regarded as a good level for that community. The ability to give an example in each pattern of abuse was used to assess knowledge. An accurate answer to each pattern of abuse was scored 1. Thus, the overall score was five. A score of 3 and above was assigned to indicate good knowledge, and a score of less than 3 was adjudged to indicate poor knowledge; thereafter, the proportion of respondents with good knowledge was estimated.

Data analysis was conducted using IBM SPSS (International Business Machines Statistical Package for the Social Sciences) Version 23, through which spreadsheets, frequency tables, and cross-tabulations were generated. Inferential statistics were used to test for association between variables using the chi-square test. Predictors of elder abuse were determined using logistic regression analysis. Statistical significance was set at p<0.05.

Ethical approval for the study was obtained from the Ethical Committee of the Irrua Specialist Teaching Hospital, Irrua, Edo State, with reference number ISTH/HREC/20171023/33. Permission was also obtained from the Chairmen of the selected local government areas and community leaders of the selected communities. Participation was entirely voluntary, and confidentiality and anonymity were assured. Verbal as well as written informed consent was obtained from the participants, and they were also informed of their right to decline or withdraw from the study at any time without consequence.

## Results

A total of 230 elderly persons were interviewed, with close to half (46.1%) between 60 and 69 years old, and a mean age of 73.2±9.1 years. There were more females, 135 (58.2%), while most of the respondents, 180 (77.6%), were retired. Approximately two-thirds, 141 (60.8%), were married. A little over a quarter of the respondents, 63 (27.2%), had primary education, a third, 77 (33.2%), had secondary education, while 68 (29.3%) had no formal education. More than two-fifths, 104 (44.8%), were living with their children or grandchildren, while the same number also reported being financially dependent on others.

The result displayed in Table 2 indicates that the majority of the respondents, 184 (79.3%), reported having been abused before, with neglect 135 (73.4%), financial abuse 118 (64.1%) and emotional abuse (53.8%) being the commonly experienced forms of abuse.

**Table 2:** Prevalence and pattern of elder abuse among the respondents

Variables	Frequency, n (%)
**Have you ever been abused?**	
Yes	184 (79.3)
No	48 (20.7)
**Type of abuse received**	
Physical abuse	72 (39.1)
Emotional abused	99 (53.8)
Neglect	135 (73.4)
Financial abuse	118 (64.1)
Sexual Abuse	1 (0.5)
**Knowledge of any abused person around you?**	
Yes	171 (73.7)
No	61 (26.3)

Table 3 shows that more than two-fifths, 81 (44.3%) perpetrators of abuse were the children of the victims, followed by house-helps/caregivers, 30 (16.3%), neighbours, 28 (15.2%) and relatives, 21 (11.4%).

**Table 3:** Perpetrators of elder abuse among the abused elderly

Perpetrators	Freq. n(%)
Spouse	9 (4.9)
Children(Married/unmarried)	81 (44.0)
Grandchildren	15 (8.2)
Neighbour	28 (15.2)
Relatives	21 (11.4)
Others (e.g. househelps, caregivers)	30 (16.3)

There is a statistically significant association between elder abuse and sex (p = 0.009), marital status (p = 0.028), employment status (p = 0.002), educational qualification (p = 0.001), living arrangement (p = 0.001), financial status (p = 0.017), and dependency level (p = 0.015). Being a female, divorced/cohabiting, unemployed, having no formal education, living with children, being dependent in activities of daily living, and partially self-sufficient financially were associated with elder abuse.

Age, sex, marital status, employment status, and educational status were the sociodemographic predictors of abuse in rural areas. Other predictors were living arrangement, financial situation, level of dependency (ADL), and having assistance at home, which were all associated with elder abuse in the rural communities (p<0.05). The odds ratio of abuse for the females (OR =2.06; CI =1.141 -4.672) shows that females are twice as likely to be abused as their male counterparts in rural communities. Divorced elderly (OR =3.435; CI =1.694 -7.789) and the widowed (OR =5.135; CI =1.093 -9.042) were about three and five times more likely to suffer from abuse than elders that are single respectively; the unemployed (OR =4.816; CI =1.134 -7.270), had a higher likelihood of approximately five times more chances of suffering an abuse than the employed; elders with no formal education were approximately three time more likely to be abused than elders with tertiary educational status (OR =2.995; CI =1.013 -4.118); living with married children (OR =2.683; CI =1.243 -5.045) and family members (OR = 3.736; CI =1.624 - 6.275) increases the chances of being abused by approximately three and four times more than those living alone respectively; being abused was about 3 times higher for the financially dependent elders than the economically independent. The chances of being abused among the dependent were approximately 3 times higher than the independent, while having assistance at home made elders in the rural communities eight times more exposed to abuse.

A majority, 142 (77.2%), of the abused victims had ever reported being abused to other people, with 94 (66.2%) of them seeking help from family members. In most cases (62.7%), no action was taken against the perpetrator. Significant barriers to help-seeking behaviour were stigmatisation 33, 24.4%), fear that reporting will worsen the abuse or the caregiver may leave (26, 19.3%).

## Discussion

This study evaluated the prevalence, pattern, and predictors of elder abuse, as well as barriers to help-seeking behaviour in two rural communities of Edo State, Nigeria. The prevalence of elder abuse found in this study was 73.4%. This implies that over two-thirds of elderly persons in the communities had been abused.

The result of this study is consistent with the findings from a study done in Imo state, which revealed that the majority (78.8%) of the elders have been abused,^14^ and in contrast to that of Okojie,^15^ who reported a prevalence of 14.7%. However, the findings are lower than what was reported from some studies in Northern Nigeria, where the burden of elder abuse was as high as 93.7% in Nasarawa state,^16^ and 100% in Kano state.^17^

The patterns of abuse in this study show that neglect is the most predominant form of abuse, followed by financial abuse and emotional abuse, with sexual abuse being the least. This finding is in tandem with studies done by Akpan in Akwa-Ibom,^18^ as well as studies in India, the USA and the UK, which portrayed neglect as the most typical type of elder abuse.^19^-^21^ However, it negates the findings from some other studies that have reported psychological/emotional abuse as the primary form of abuse.^14^-^17^ Another study reported mixed findings of neglect among elderly women and psychological abuse among men.^17^ Generally, the possible reasons for the high prevalence of elder abuse may include: breakdown of the extended family system, near collapse of our social support system and the quest for better life and greener pastures, which has led to mass rural-urban migration of many young adults.^4^ The high pattern of elder abuse due to neglect and financial issues is more worrisome and hazardous for elders in developing countries like Nigeria, where the standard of living is very low and in which the social welfare support system is absent for the elderly.

Several studies have reported sexual abuse as the least common type of abuse,^21^,^22^ and this was corroborated by our finding of only one case of sexual abuse in an elderly female, in the rural community who had not only been abused sexually by the strangers, but had resorted to transactional sex as a means of livelihood due to neglect by her family members.

According to the findings of this study, the majority of the perpetrators of abuse were children of the victims and family members. Adult children are the most likely to extort pensions, steal property, blackmail, neglect, and physically, psychologically, and sexually abuse their elders.^6^,^23^ Other studies of elder mistreatment also indicate that family members are the most frequent perpetrators of elder abuse,^19^ with the perpetrators usually adult children, grandchildren, and other relatives; professional caregivers; and close friends or others in a position of trust.^24^-^26^

This study's findings show that being a female, divorced, unemployed, having no formal education, living with married children, being dependent at activities of daily living and partially self-sufficient financially were significant predictors of elder abuse. This finding is similar to those of a study conducted by Ibrahim in Awe, Nasarawa State, and Ekot in Akwa Ibom State, Nigeria.^16^,^26^ Contrary to reports by other studies that elder abuse is not gender specific, with no association between gender and elder abuse, as both genders are equally susceptible,^20^,^27^ this study found that being female was significantly associated with elder abuse, which is similar to reports from some previous studies.^16^,^22^ Plausible explanations are increased vulnerability and inability to defend oneself, increased financial dependency emanating from the inferior status accrued to the girl child by the society, as well as losing the right to inheritance and lower opportunities to education and wealth creation in a male-dominated environment.

This study identified that living with married children and other family members is a risk factor for abuse, with a three- and four-time risk, respectively, than those living alone. This is similar to findings from studies in Bangladesh, Israel and New York, which reported that living with married children and other family members was significantly associated with abuse.^28^-^30^ It is however in contrast with studies done in Oyo and Akwa Ibom state, which reported that living alone is a risk factor for abuse^21^,^31^ as well as in comparison to a study by Perez et al which found no relationship, nevertheless, they attributed abuse to possible dysfunctional relationship existing between the victim and the perpetrator.^32^

Regarding help-seeking behaviour, this study found that the elderly exhibit good help-seeking behaviour, with help primarily sought from family members and community leaders, rather than formal support services, which were used by only a few.

This is in tandem with a study in the USA,^33^ that reported that help-seeking through reporting to police or other authorities was low among elderly abused victims, and mainly occurred among victims of physical abuse and poly-victimisation.

Regarding barriers to non-reporting, no respondent in the rural area claimed ignorance of who or where to seek help in the event of abuse, which may imply that rural dwellers have good neighbourliness and community support. However, this study reported that the significant barriers to help-seeking were fear of stigmatisation, scandalisation of offsprings (as perpetrators of abuse mainly were the victims' children and other family members), fear that the abuse may worsen, fear of a strained relationship with the caregiver and that the perpetrator to who they are dependent upon for care may leave.^34^,^35^

The estimated prevalence from this study may be underreported because most elderly persons are reluctant to fully disclose relevant information on issues of abuse due to various factors. However, this was addressed by conducting private and confidential interviews with the elderly. Also, this study was conducted among the cognitively intact elderly, as well as those with no communication difficulties. Thus, abuse among the cognitively impaired and severely ill elderly, who are mostly thought to be the group at most significant risk of abuse, was not assessed.

## Conclusion

In conclusion, elder abuse is highly prevalent in this study, with neglect as the most typical form of abuse, and children of the victims are the major perpetrators. The significant risk factors for elder abuse in this study are; being a female, divorced/cohabiting, unemployed, having no formal education, living with married children, being dependent at activities of daily living and partially self-sufficient financially.

Against this background, the following recommendations are made: The United Nations and other relevant organisations should intensify efforts on the promotion of World Elder Abuse Awareness Day (June 15 of every year), while the government at all levels (Federal, State and Local) should implement sustainable policies and interventions including enlightenment and education, provision of social safety nets and healthcare services. The importance of relevant interventions at the community and family level, especially concerning respect and care for the elderly, cannot be overemphasised.

## Figures and Tables

**Table 1:** Socio-demographic Characteristics of Respondents (n = 232)

Socio-demographics	Frequency, n (%)
**Age (years)**	
Old elderly (60-69)	107 (46.1)
Older elderly (70-79)	62 (26.7)
Oldest elderly (80 and above)	63 (27.2)
Mean age	73.2±9.1
**Sex**	
Male	97 (41.8)
Female	135 (58.2)
**Religion**	
Christianity	171 (73.7)
Islam	32 (13.8)
Traditional	25 (10.8)
Others	4 (1.7)
**Employment Status**	
Retired	180 (77.6)
Employed	52 (22.4)
**Marital status**	
Single	7 (3.0)
Married	141 (60.8)
Divorced	14 (6.0)
Widowed	64 (27.6)
Cohabiting	6 (2.6)
**Educational Status**	
No formal education	68 (29.3)
Primary school	63 (27.2)
Secondary school	77 (33.2)
Tertiary	24 (10.3)
**Living arrangement**	
Living alone	24 (10.3)
Living with spouse	78 (33.6)
Living with child(ren)/grandchild(ren)	104 (44.8)
Living with married child(ren)	34(14.7)
**Living with other family members**	26 (11.2)
**Living with paid caregiver**	0 (0.0)
**Dependency Level**	
**Totally dependent**	18 (7.8)
**Partially dependent**	125 (53.9)
**Independent**	89 (38.4)
**Financial Status**	
**Financial self-sufficient**	41 (17.7)
**Partially self-sufficient**	87 (37.5)
**Total financial dependence**	104 (44.8)

**Table 4:** Factors associated with elder abuse

Variables	Abused (N=184)	Never abused (N=48)	χ^2^	P-value
**Age**			2.533	
Old Elderly	80 (74.8)	27 (25.2)		0.282
Older Elderly	52 (83.9)	10 (16.1)		
Oldest Elderly	52 (82.5)	11 (17.5)		
**Sex**				
Male	69 (71.1)	28 (28.9)	6.791	0.009*
Female	115 (85.2)	20 (14.8)		
**Marital status**				
Single	4 (57.1)	3 (42.9)	10.906	0.028*
Married	115 (81.6)	26 (18.4)		
Divorced	14 (100.0)	0 (0.0)		
Widowed	45 (70.3)	19 (29.7)		
Cohabiting	6 (100)	0 (0.0)		
**Employment Status**				
Retired	152 (84.4)	28 (15.6)	12.913	0.002*
Employed	32 (61.5)	20 (38.5)		
**Educational qualification**				
No formal education	65 (95.6)	3 (4.4)	15.671	0.001*
Primary education	45 (71.4)	18 (28.6)		
Secondary education	56 (72.7)	21 (27.3)		
Tertiary education	18 (75.0)	6 (25.0)		
**Living arrangement status**				
Living alone	10 (41.7)	14 (58.3)	34.603	0.001*
Living with spouse	60 (77.0)	18 (23.0)		
Living with unmarried child(ren)	12 (66.7)	6 (33.3)		
Living with married child(ren)	34 (100.0)	0 (0.0)		
Living with grandchild(ren)	45 (86.5)	7 (13.5)		
Living with other family members	23 (88.5)	3 (11.5)		
Living with paid caregiver	0 (0.0)	0 (0.0)		
**Financial status**				
Financially self sufficient	26 (63.4)	15 (36.6)	8.199	0.017*
Partially self-sufficient	74 (85.1)	13 (14.9)		
**Dependency level**				
Totally dependent	16 (88.9)	2 (11.1)	8.350	0.015*
Partially dependent	106 (85.0)	19 (15.0)		
Independent	62 (69.7)	27 (30.3)		

* *P-value is less than 0.05 alpha level and significant*

**Table 5:** Predictors of elder abuse

					95% C.I. for Exp (B)	
	B	S.E.	Sig.	Odd ratio	Lower	Upper
**Age**	1.275	.458	.022*	3.579	.950	5.940
**Sex**	-.723	.231	.002*	2.061	1.141	4.672
**Marital status**						
Marital status (Divorced)	-1.234	.466	.027*	3.435	1.694	7.789
Marital status (Widowed)	-1.636	.431	.000*	5.135	1.093	9.042
**Employment status**						
Employment status (Unemployed)	-1.572	.424	.000*	4.816	1.134	7.270
**Educational status**						
Educ. qual (no educ.)	-1.097	.309	.000*	2.995	1.013	4.118
**Living arrangement**						
Living with married children	.987	.352	.027*	2.683	1.243	5.045
Living with family members	-1.318	.429	.020*	3.736	1.624	6.275
**Financial status**						
Financially dependent	1.209	.481	.042*	3.350	1.164	7.322
**ADL Class**						
ADL Class (totally dependent)	1.091	.353	.010*	2.977	1.069	5.037
**Do you have assistance at home (Yes)**	-2.133	.856	.042*	8.440	3.190	12.225

**Table 6:** Help Help-seeking behaviour among abused elders

Help seeking behaviour	Frequency, n (%)
**Have you ever reported?**	
Yes	142 (77.2)
No	42 (22.8)
**To whom did you report to?**	
Family member(s)	94 (66.2)
Community leader	30 (21.1)
Police	1 (0.7)
**Others	17 (12.0)
**Was any action taken**	
Yes	53 (37.3)
No	89 (62.7)
**Reason for not reporting**	
I don't know who/where to go to	0 (0.0)
I fear reporting will worsen the abuse	26 (19.3)
I am scared cause of stigmatisation, so would rather keep quiet	33 (24.4)
Reporting may make the caregiver go away, thus leaving me totally unattended	26 (19.3)
I am not sure the issue will be addressed	16 (11.9)
Its culturally wrong to report your ward	22 (16.3)
I think it's karma	10 (7.4)
I have no financial capability to seek redress	2 (1.5)

## References

[1] WHO (2008). A Global response to elder abuse and neglect: Building primary health care capacity to deal with the problem worldwide.

[2] Lachs MS, Pillemer KA (2015). Elder Abuse. N Engl J Med.

[3] WHO (2002). The Toronto declaration on the global prevention of elder abuse.

[4] WHO (2017). Elder abuse.

[5] Daramola OE, Awunor NS, Akande TM (2018). The Challenges of Retirees and Older Persons in Nigeria; a Need for Close Attention and Urgent Action. Int J Tropical Disease & Health.

[6] Cadmus EO, Owoaje ET, Akinyemi OO (2015). Older persons' views and experience of elder abuse in South Western Nigeria: a community-based qualitative survey. J Aging Health.

[7] Dong XQ (2015). Elder abuse: Systematic review and implications for practice. J Am Geriatr Soc.

[8] Abdel-Rahman T, El Gaafary M (2012). Elder mistreatment in a rural area in Egypt. Geriatr Gerontol Int.

[9] World Health Organization (2002). Missing voices: views of older persons on elder abuse.

[10] (2014). Elder abuse.

[11] Edo State Government of Nigeria (2017). Edo people.

[12] National Population Commission (NPopC) Nigeria (2010). 2006 Population and Housing Census. Vol. IV.

[13] Cochran WG (1977). Sampling techniques.

[14] Oluoha RU, Obionu CN, Uwakwe KA, Diwe KC, Duru CB, Merenu IA (2017). Assessing the Prevalence and Patterns of Elder's Abuse in Imo State, Nigeria: A Rural – Urban Comparative Study Assessing the Prevalence and Patterns of Elder's Abuse in Imo State, Nigeria: A Rural – Urban Comparative Study. JAMPS.

[15] Okojie OH, Omuemu VO, Uhunwangho JI (2022). Prevalence, Pattern and Predictors of Elder Abuse in Benin City, Edo State, Nigeria: An Urban and Rural Comparison. West Afr J Med.

[16] Ibrahim AH, Ibrahim AH, Ladan MA, Abubakar FU, Garba SN, Musa-Maliki AU (2022). Prevalence and Pattern of Elder Abuse in Awe, Nasarawa State, Nigeria. GIJMSS.

[17] Mustapha NM (2017). Nature and Prevalence of Elderly Abuse and Neglect in Kano Municipal Local Government Area of Kano State, Nigeria. GOJAMSS.

[18] Akpan ID, Umobong ME (2013). An Assessment of the Prevalence of Elder Abuse and Neglect in Akwa Ibom State, Nigeria. Dev Ctry Stud.

[19] Chandanshive P, Subba SH, Parida SP, Mishra S (2022). Prevalence patterns and associated factors of elder abuse in an urban slum of eastern India. BMC Geriatr.

[20] Acierno R, Hernandez MA, Amstadter AB, Resnick HS, Steve K, Muzzy W (2010). Prevalence and correlates of emotional, physical, sexual, and financial abuse and potential neglect in the United States: The national elder mistreatment study. Am J Public Health.

[21] Biggs S, Manthorpe J, Tinker A, Doyle M, Erens B (2009). Mistreatment of Older People in the United Kingdom: Findings from the First National Prevalence Study. J Elder Abuse Negl.

[22] Cadmus E, Owoaje E (2012). Prevalence and correlates of elder abuse among older women in rural and urban communities in south western Nigeria. Heal Care Women Int.

[23] Teaster PB, Roberto KA, Geffner R, White JW, Hamberger LK, Rosenbaum A, Vaughan-Eden V, Vieth VI (2022). Perpetrators of Elder Abuse. Handbook of Interpersonal Violence and Abuse Across the Lifespan.

[24] Karina PL Perpetrators of elder abuse mostly adult children.

[25] Sonia S Elder abuse: Who are the perpetrators?.

[26] Ekot M (2015). Societal factors in elder abuse in Akwa Ibom State. Int J Home Econ.

[27] Strasser S, Smith M, Weaver S, Zheng S, Cao Y (2013). Screening for Elder Mistreatment among Older Adults Seeking Legal Assistance Services. West J Emerg Med.

[28] Munsur AM, Tareque I, Rahman KM (2010). Determinants of living arrangements, health status and abuse among elderly women: A study of rural Naogaon District, Bangladesh. J Int Womens Stud.

[29] Burnes D, Pillemer K, Caccamise PL, Mason A, Henderson CR, Berman J (2015). Prevalence of and Risk Factors for Elder Abuse and Neglect in the Community: A Population-Based Study. J Am Geriatr Soc.

[30] Lowenstein A (2010). Caregiving and Elder Abuse and Neglect-Developing a New Conceptual Perspective. Ageing Int.

[31] Igbokwe CC, Asogwa LO (2010). Prevalence of abuse of the elderly in domestic setting in Enugu state, Nigeria. J Home Econ Res.

[32] Pérez-Rojo G, Izal M, Montorio I, Penhale B (2009). Risk factors of elder abuse in a community dwelling Spanish sample. Arch Gerontol Geriatr.

[33] Burnes D, Acierno R, Hernandez-Tejada M (2019). Help-Seeking Among Victims of Elder Abuse: Findings from the National Elder Mistreatment Study. J Gerontol B Psychol Sci Soc Sci.

[34] Chane S, Adamek M (2015). Death is better than misery: Elders' accounts of abuse and neglect in Ethiopia. Int J Aging Hum Dev.

[35] Qu L, Kaspiew R, Carson R, Roopani D, De Maio J, Harvey J (2021). National Elder Abuse Prevalence Study: Summary Report. Research Report.

